# Lacticaseibacillus casei JS-2 from ‘Jiangshui’ Reduces Uric Acid and Modulates Gut Microbiota in Hyperuricemia

**DOI:** 10.3390/foods14030407

**Published:** 2025-01-26

**Authors:** Jiahui Wu, Xiang Wang, Lvbu Aga, Leimengyuan Tang, Shuting Tan, Dachuan Zhang, Houxier Li, Li Yang, Nan Zhang, Shiyao Su, Maochun Xiao, Rongting Min, Aji Li, Xueyong Wang

**Affiliations:** 1 School of Chinese Meteria Medica, Beijing University of Chinese Medicine, Northeast Corner of Intersection of Sunshine South Street and Baiyang East Road, Fang-Shan District, Beijing 102488, China; 2 Bayingolin Mongolian Autonomous Prefecture Institute for Food and Drug Control, No. 101, North Jianguo Road, Korla City 841000, Bayingolin Mongol Autonomous Prefecture, Xinjiang Uygur Autonomous Region, China

**Keywords:** hyperuricemia, gout, uric acid, gut microbiota, JS-2

## Abstract

Lacticaseibacillus casei (JS-2) is a novel probiotic isolated from “Jiangshui”, a kind of traditional folk fermented food, which has a significant effect on hyperuricemia (HUA). In vitro experimental results showed that JS-2 has a high degradation ability and selectivity for uric acid (UA). The animal test results indicated that after two weeks of treatment, JS-2 could significantly reduce the level of UA in the serum of HUA quails (*p* < 0.01), and its effect is almost equivalent to that of the positive drug control group, benzbromarone. Further, after JS-2 treatment, the level of xanthine oxidase in quail serum decreased significantly. Analysis data of quail fecal metabolomics results showed that JS-2-altering metabolites were involved in amino acid, purine, and lipid metabolism. To investigate the mechanism underlying JS-2-mediated UA degradation in the quail model of HUA, 16S rRNA gene sequencing was conducted. It was found that the structure and function of the gut microbiota were restored after JS-2 intervention, and the abundance of short-chain fatty acid (SCFA)-producing bacteria (g__Ruminococcus_torques_group and g__Butyricicoccus) and bacteria with UA degradation capacity (g__unclassified_f__Lachnospiraceae and g__Negativibacillus) increased significantly; intestinal SCFAs, especially propionic acid, increased accordingly. These experimental data suggest that the beneficial effects of JS-2 may derive from changes in the gut microbiome, altering host–microbiota interactions, reducing UA levels by increasing UA excretion, and reducing absorption. These findings provided new evidence that JS-2 has the potential to be used as a naturally functional food for the prevention of HUA.

## 1. Introduction

Hyperuricemia (HUA), an idiopathic inflammatory disorder, is manifested by persistent derangements in purine metabolism and a continual elevation in the blood uric acid (UA) concentration. In the absence of uricase within the human body, uric acid fails to be efficiently transformed into water-soluble allantoin, thereby hindering its smooth elimination from the body [1,2,3].

In the past two decades, the worldwide prevalence of hyperuricemia and gout has increased twofold [4]. It has been reported that the prevalence rates among different populations worldwide range from 2.6% to 36% [5]. In China, the number of hyperuricemia patients has reached 170 million, with approximately 47% progressing to gout, and the condition shows an annual growth rate of 9.7% [6].

The rapid increase in its incidence has become a significant threat to human health [7]. A diet rich in purines and an unhealthy lifestyle can disrupt UA homeostasis, leading to an elevation in plasma UA levels and increasing the risk of HUA [8]. HUA may result in UA crystal deposition, increasing the burden of comorbidities and ultimately leading to damage such as gout [9], atherosclerosis [10], and type 2 diabetes [11]. The treatment principle for hyperuricemia aims to inhibit UA accumulation. UA homeostasis is maintained by its synthesis in the liver and its elimination via the kidneys and intestines. The most commonly used anti-hyperuricemic drugs clinically include allopurinol, benzbromarone (uricosuric) [12], and uricase (urate oxidase) [13]. However, they all have adverse effects, such as side effects like changes in dietary habits, liver function impairment, and gastrointestinal reactions [8]. Given the limitations of current therapeutic drugs, there is an urgent need to develop an effective and safe treatment regimen.

Probiotics are active microorganisms that confer beneficial health effects on the host and significantly alleviate metabolic syndromes, including diabetes [14] and chronic kidney disease (CKD) [15]. The gut microbiota is now recognized as a virtual metabolic organ that influences the gastrointestinal, liver, and cardiovascular systems, regulates the immune system, and prevents pathogen invasion. It affects uric acid balance by metabolizing purine substances and regulating uric acid excretion, and reabsorption processes. Purine metabolism is a major source of uric acid production. The gut microbiota can directly affect uric acid levels by regulating key enzymes (such as xanthine oxidase) and related metabolic pathways. For example, shiga-toxigenic *E. coli* (STEC) and enteropathogenic *E. coli* (EPEC) can participate in the key steps of converting hypoxanthine into uric acid, while also improving the expression of uric acid transport channels through the production of short-chain fatty acids [16,17,18]. Furthermore, studies have shown that hyperuricemia induced by a high-fructose diet leads to a reduced abundance of Bacteroides and an increased abundance of Proteobacteria and Verrucomicrobia [19]. HUA alters the structure, composition, and permeability of the gut microbiota [20]. Probiotic intervention, however, can restore gut dysbiosis induced by hyperuricemia and purine-rich diets [19], improve serum uric acid [21], and degrade uric acid. Studies have demonstrated that oral administration of fermented Limmosilactobacillus fermentum JL-3 can restore the abundance of the gut microbiota [22]. Chlorogenic acid enhances the abundance of short-chain fatty acid-producing bacteria, such as Bacteroides, in the mouse intestine, modulates gut microbiota and glutamate metabolism, and alleviates the symptoms of hyperuricemia [23]. Chicory intervention in a hyperuricemic quail model significantly reduces serum uric acid levels by increasing the abundance of probiotics (Bifidobacterium) and inhibiting the Toll-like receptor 4 (TLR4) inflammatory response, thereby alleviating hyperuricemic symptoms. Inulin alleviates HUA by restoring gut microbiota balance and suppressing the inflammatory response of the LPS/TLR4 axis in a quail model [24]. JL-3 degrades intermediate products of purine metabolism, thereby reducing uric acid levels [22]. In summary, probiotics can effectively degrade UA and enhance the activity of the human gut microbiota. JS-3 can regulate host–microbiome interactions, enhance gut UA degradation, reduce SUA and fecal UA levels, and treat hyperuricemia.

Fermented foods have always been a primary source for isolating lactic acid bacteria. “Jiangshui” is a traditional acidic fermented food from northwest China, made by first cooking and heating vegetables, then adding noodle soup and a starter culture (“old Jiangshui”) for natural fermentation. It contains various beneficial bacteria. Lactobacillus and yeast are the main microorganisms during the fermentation process [25]. This study aims to screen the probiotic JS-2 with uric acid degradation ability from the fermented “Jiangshui”, establish a purine-induced hyperuricemia quail model to assess the uric acid-lowering activity of JS-2, and explore its mechanism of action in improving the composition of the gut microbiota and hyperuricemia to provide new treatment strategies for patients.

## 2. Materials and Methods

### 2.1. Strains with UA Degradation Ability

We isolated one probiotic strain from Traditional Jiangshui samples in a UA-selective medium [22]. We transferred the selected strains to a new medium with UA as the only source of carbon and nitrogen and incubated the strain-containing medium at 37 °C for 24 h while the JS-2 single colonies were streaked on MRS agar plates to obtain individual colonies. The strain sample on the plate was smeared on glass slides to perform the Gram-stain test. After the application step, the coverslip was fixed, the section was treated with Gram-stain, and observation and analysis were carried out with a medical microscope. Then, artificial intestinal fluid and gastric juice were simulated to identify the strain characteristics. Finally, the bacterial solution was stored in 25% glycerol at −80 °C.

### 2.2. Determination of the Ability of JS-2 Strain to Degrade UA

To assess the UA-degrading ability of JS-2 strains, JS-2 was inoculated into MRS medium and incubated under anaerobic conditions for 24 h. It was centrifuged at 600× *g* for 15 min, washed twice and incubated at 37 °C. Samples were taken at 24, 48, and 72 h. The solution was centrifuged at 600× *g* for 15 min and the supernatant was discarded. After washing with sterile water, 20 μL of HClO_4_ was added and a kit was used to measure UA. The changes in UA content were calculated.

### 2.3. Analysis of Strain Gene Sequencing

Canna edulis starch was purchased from TIANGEN Co., Ltd. (Beijing, China). Genomic DNA of the strain was extracted using a TIANamp bacterial DNA kit, and the PCR product was sent to Sangon Biotech (Shanghai, China) Co., Ltd. for sequencing. The species were identified by comparing the alignment sequence with the BLAST Engine (NCBI). The morphology of JS-2 was examined through scanning electron microscopy.

### 2.4. Experimental Design Based on Animal Models

A quail model of HUA was established through the combined administration of yeast and adenine [26]. The normal group was fed ordinary quail feed, and the modular group was fed with model feed. We stirred yeast extract powder (into ordinary quail feed, and the feeding amount of each quail yeast was calculated as 20% yeast feed 15 g/(kg·d) [25], and ordinary feed was supplemented after eating. The animals were allowed to drink freely. Yeast extract powder was purchased from Oxoid Co., Ltd. (Basingstoke, UK). SUA content was measured two weeks after modeling to model HUA. They were grouped according to body weight, with eight birds in each group. The JS-2 group was given gavage with a daily bacterial volume of 2 × 10^8^ colony-forming units, the control group was free to drink water and the positive group was treated with benzbromarone 20 mg/(kg·d) Benzbromarone was purchased from Hermann Pharma (London, UK).

### 2.5. Determination of SUA Content

Frozen quail feces were placed in a 60 °C water bath for 15 min, and then vortexed for homogenization. The solution was collected by centrifugation at 1000× *g* for 8 min. A UA detection kit was used to measure UA content, and fluorescence was analyzed at a wavelength of 564 nm to determine the UA levels in serum samples. UA detection kit was purchased from Zhongsheng Beikong Biotechnology Co., Ltd. (Beijing, China).

### 2.6. Fecal Sample Analysis: Determination of SCFAs in Quail Feces

2-ethylbutyric acid solution was purchased from Macklin Co., Ltd. (Shanghai, China). Eppendorf tubes and 0.22 μm microporous membrane were purchased from LABLEAD Co., Ltd. (Beijing, China). We took 1 g of fresh feces and 2-ethylbutyric acid solution as the internal standard, and 5 mL of an aqueous solution containing the internal standard was added, adjusting the pH with formic acid (pH = 2–3). The solution was mixed evenly, shaken, and centrifuged (4 °C, 10,000× *g*, 15 min). We transferred the supernatants to new Eppendorf tubes, filtered them through a 0.22 μm microporous membrane, and injected it into a GC (Agilent 7890A) for analysis. N_2_ was the vehicle with an injection volume of 1 μL; the concentration of SCFAs in the supernatant was measured with a column (DB-WAX, 30 mm × 0.32 mm × 0.5 μm). We calculated the SCFA content in each sample. 

### 2.7. Serum Biochemical Analysis and Histopathological Evaluation of Liver and Kidney

Quail serums were taken, and CRE and BUN were measured using an automatic biochemical analyzer (Beckman Coulter, CA, USA). A kit was used to measure XOD (Nanjing, China). The kidney samples were stained with hematoxylin/eosin.

### 2.8. 16S rRNA Amplification

On day 14 after starting treatment, feces and cecal contents were collected. High-quality genomic DNA was required depending on the sample type. Fragments were generated using the standard operating procedure of the Illumina MiSeq platform (Illumina, San Diego, CA, USA). OTU clustering analysis and species taxonomic analysis were performed.

### 2.9. Fecal Metabolomics Studies After JS-2 Intervention

#### 2.9.1. Collection and Preparation of Samples

We used 60 mg of fecal samples, placed them in 2 mL Eppendorf tubes, and then added 600 μL of 80% methanol. The samples were pre-cooled at −20 °C, ground, and sonicated in an ice-water bath. After that, they were allowed to stand still for 30 min before being centrifuged. The supernatant was collected, filtered, and used to prepare the quality control samples.

#### 2.9.2. Metabolomic Data Analysis

Data processing: raw data from mass spectrometry analysis were pre-processed using SIEVE and MetaboAnalyst 5.0 software. The processed data were analyzed using the Xcalibur 4.2 workstation with HMDB. Each group of stool samples was imported into SIMCA software (14.1), clustered by PCA. OPLS-DA looks for groups of potential biomarkers.

### 2.10. Statistical Analysis

The data were analyzed with GraphPad Prism 8.0.2, using a one-way analysis of variance (ANOVA) to compare means. Between-group comparisons were used for nonparametric tests, and *p* < 0.05 indicated statistical significance.

## 3. Results

### 3.1. Screening, Isolation, and Identification of UA-Degrading Strains from “Jiangshui”

**Figure 1:**
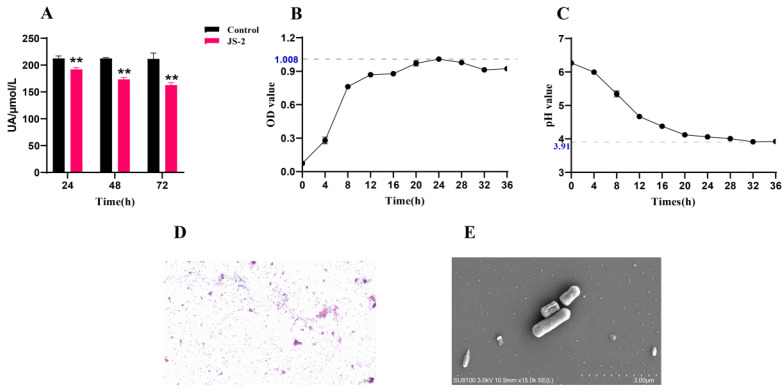
Screening, identification, and characterization of the JS-2 strain from “Jiangshui”. (**A**) The ability of the JS-2 strain to degrade UA in vitro within 24 h, 48 h, and 72 h. Control: A sample containing a specific concentration of UA, used as a reference. (**B**) The growth curve of JS-2 within 36 h. (**C**) Changes in the pH value of JS-2 in UA medium. (**D**) Gram-stain results of strain JS-2 (200× magnification). (**E**) Scanning electron microscope morphology of JS-2. * *p* < 0.05, ** *p* < 0.01 compared with the control group. Data are shown as means ± SD (*n* = 8).

To screen for probiotics that reduce uric acid (UA), we used a selective culture method using UA as the sole source of carbon and nitrogen in a commercial fermentation medium. From untreated Chinese fermented “Jiangshui”, we successfully isolated a probiotic strain (JS-2) capable of degrading UA. JS-2 exhibited significant UA degradation, reducing UA by 9.64%, 18.36%, and 23.05% at 24 h, 48 h and 72 h (Figure 1A). Furthermore, JS-2 also demonstrated excellent growth characteristics (reaching peak growth at 24 h) and acid production ability (with the medium’s pH reaching its lowest point at 32 h, dropping by two orders of magnitude from the initial value) (Figure 1B,C). We identified JS-2 and found it to be a G+ bacterium (Figure 1D). Based on the NCBI BLAST database analysis, the JS-2 strain showed a close genetic relationship to Lacticaseibacillus casei (L. casei). Transmission electron microscopy (TEM) further confirmed that the typical morphology of JS-2 is consistent with that of L. casei (Figure 1E). These findings indicate that JS-2 is a strain of L. casei with the potential to degrade UA.

### 3.2. JS-2 Lowered Serum Uric Acid Levels and Alleviated Liver and Kidney Damage in Hyperuricemic Quails

**Figure 2:**
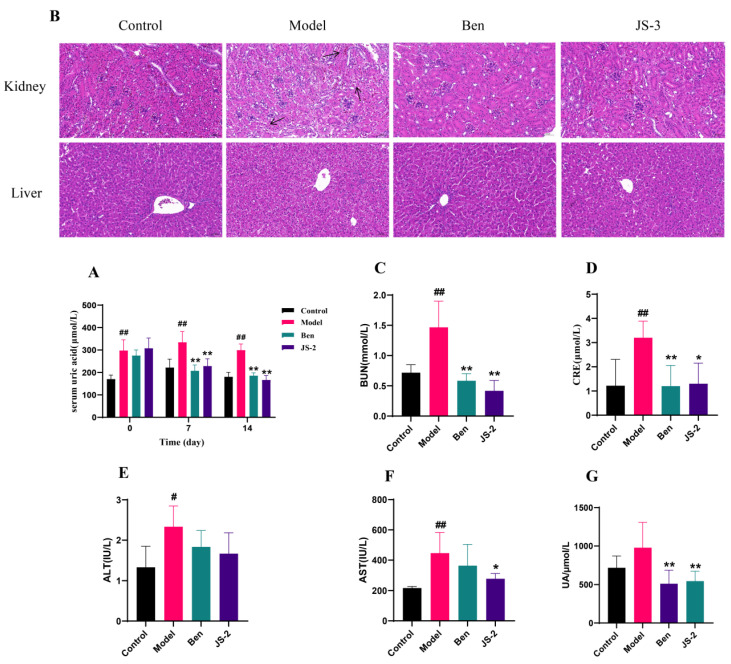
The impact of JS-2 on quails with hyperuricemia. (**A**) The level of SUA. (**B**) Representative micrographs of H&E-stained kidney and liver tissues of quails (200× magnification). Arrows: cytoplasmic vacuolization. (**C**–**F**) Levels of BUN, CRE, ALT, and AST in quails. (**G**) The UA degradation ability of the gut microbiota in quails. # *p* < 0.05, ## *p* < 0.01 compared to the control group, * *p* < 0.05, ** *p* < 0.01 compared to the model group. Data are presented as means ± SD (*n* = 8 per group).

In vitro experiments showed that JS-2 is capable of degrading uric acid (UA) and maintaining high survivability in the gastrointestinal tract (Table S1). To further assess whether JS-2 can reduce serum uric acid (SUA) levels, we used quails, which, like humans, lack xanthine oxidase (XO), to establish a hyperuricemia model. The model group quails exhibited significantly higher SUA levels compared to the control group. Following interventions with JS-2 and benzbromarone, SUA levels significantly decreased, with JS-2 showing superior efficacy compared to benzbromarone (Figure 2A). Notably, quails with hyperuricemia exhibited significant liver damage (mild hepatic steatosis and vacuolar degeneration) and kidney damage (inflammatory cell infiltration, tubular epithelial cell degeneration or necrosis with nuclear pyknosis or karyolysis and cytoplasmic vacuolization, and mild interstitial edema were observed). These signs of liver and kidney damage induced by hyperuricemia were significantly improved after intervention with JS-2 and benzbromarone (Figure 2B). Similarly, liver function indicators [alanine aminotransferase (ALT) and aspartate aminotransferase (AST)] and kidney function markers [blood urea nitrogen (BUN) and creatinine (CRE)] were evaluated in each group of quails (Figure 2C–F). The levels of ALT, AST, BUN, and CRE were significantly elevated in quails with hyperuricemia. After the intervention, the JS-2 and Ben groups showed significant improvements in liver and kidney functions, with notable reductions in BUN and CRE levels, and JS-2 significantly decreased AST levels. Fecal samples were also collected from the various groups of quails. After 24 h of cultivation in the GAM medium, the model group exhibited an increase in UA levels, while the UA levels in the JS-2 group were reduced by 44.17% (Figure 2G). This result indicated that the gut microbiota in the JS-2 group had a significant ability to degrade UA. These revelations signify that JS-2 is capable of effectively diminishing SUA levels in hyperuricemic quails, along with alleviating hepatic and renal impairments, and its functionality is tightly intertwined with the gut microbiota. Additionally, in contrast to the positive control drug benzbromarone, JS-2 manifested a more preponderant efficacy.

### 3.3. JS-2 Regulated Gut Microbiota Dysbiosis in Hyperuricemic Quails

**Figure 3:**
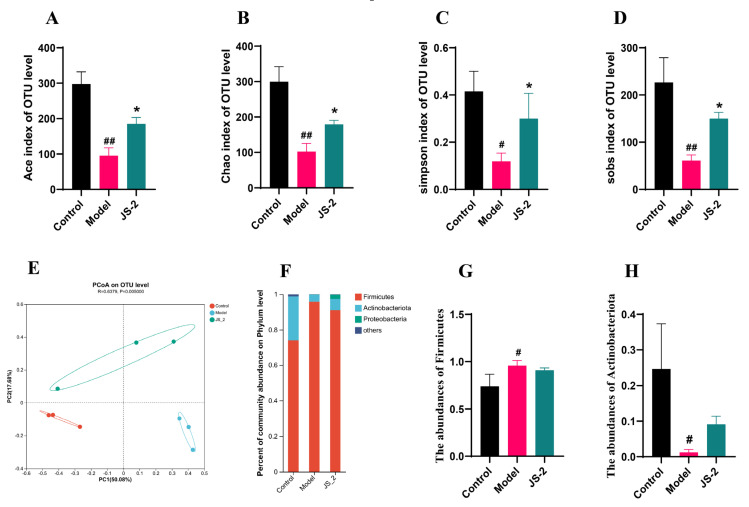
The effect of JS-2 treatment on the gut microbiota diversity of hyperuricemic quail. Microbial *α*-diversity analysis of (**A**) ace, (**B**) chao, (**C**) simpson and (**D**) sobs. (**E**) UniFrac-based PCoA analysis. (**F**) Phylum-level composition of the gut microbiota. The abundance of (**G**) Firmicutes and (**H**) Actinobacteriota. # *p* < 0.05, ## *p* < 0.01, * *p* < 0.05, ** *p* < 0.01 compared with the model group. Data are shown as means ± SD (*n* = 3).

**Figure 4:**
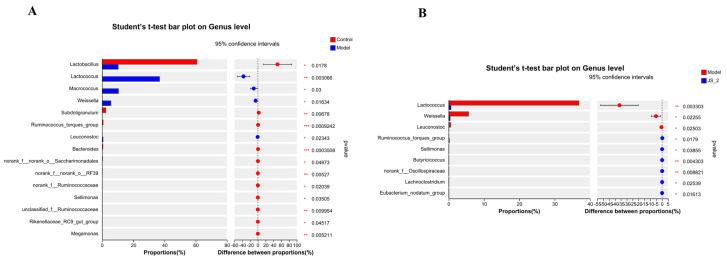
The effect of JS-2 treatment on the composition of gut microbiota in quails with hyperuricemia. One-way ANOVA analysis of (**A**) control vs. model groups and (**B**) model vs. JS-2 groups. * *p* < 0.05, ** *p* < 0.01, *** *p* < 0.01. Data are shown as means ± SD (*n* = 3, each group).

MiSeq sequencing analysis of 16S rRNA was conducted to examine changes in the gut microbiota of hyperuricemic quails treated with JS-2. The sequencing data volume is reasonable and sufficient to cover “all” microorganisms present in the samples (Figure S1). *α*-diversity analysis revealed that the richness and diversity of the gut microbiota in hyperuricemic quail were significantly reduced. However, after JS-2 intervention, both richness and diversity significantly increased (Figure 3A–D). To examine the similarities or differences in overall community structure among the healthy, diseased, and treatment groups, a principal coordinates analysis (PCoA) plot was generated using unweighted UniFrac. The PCoA plot (Figure 3E) shows a significant separation between the control group and the model group along the PC1 axis, highlighting a marked alteration in the gut microbiota structure of hyperuricemic quails. The JS-2 group and model group are significantly separated along the PC2 axis, tending to approach the control group, suggesting that JS-2 ameliorated the gut dysbiosis associated with hyperuricemia. Firmicutes, Actinobacteriota, and Proteobacteria constituted 99% of the gut microbiota structure in hyperuricemic quail, with Firmicutes and Actinobacteriota being predominant (Figure 3F). Relative to the control group, the model group exhibited a significant increase in Firmicutes abundance and a marked decrease in Actinobacteriota abundance. After JS-2 intervention, the gut microbiota dysbiosis was partially restored (Figure 3G,H). Subsequently, we conducted a one-way ANOVA on the gut microbiota of mice to identify differential characteristic microbial taxa. The results revealed that *Lactobacillus*, *Subdoligranulum*, *Ruminococcus_torques_group*, and *Bacteroides* were the dominant genera in the control group, whereas *Lactococcus*, *Macrococcus*, *Weissella*, and *Leuconostoc* were characteristic genera in the model group (Figure 4A). Post-JS-2 intervention, there was a significant reduction in the abundance of *Lactococcus*, *Weissella*, and *Leuconostoc*; conversely, *Ruminococcus_torques_group*, *Sellimonas*, *Butyricicoccus*, *norank_f_Oscillospiraceae*, *Lachnoclostridium*, and *Eubacterium_nodatum_group* significantly increased, characterizing the JS-2 group (Figure 4B). These results indicate that JS-2 effectively modulates the gut microbiota structure in hyperuricemic quails by correcting microbial dysbiosis, increasing microbial community diversity, promoting the growth of beneficial bacteria—particularly those producing short-chain fatty acids (SCFAs)—and reducing the abundance of harmful bacteria.

### 3.4. JS-2 Regulated the Levels of SCFAs in the Intestines of Quails with Hyperuricemia

**Figure 5:**
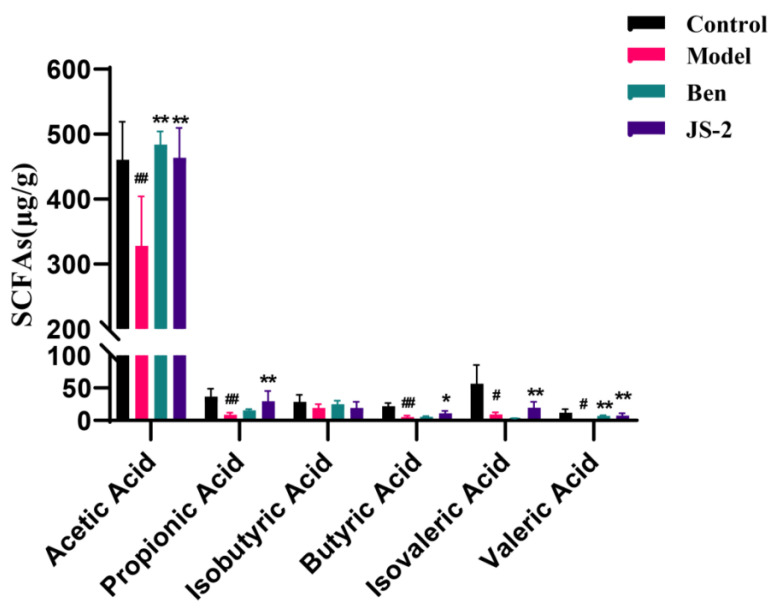
SCFA content of quails in each group. # *p* < 0.05, ## *p* < 0.01, * *p* < 0.05, ** *p* < 0.01 compared with the model group. Data are shown as means ± SD (*n* = 3).

The gut microbiota of quails with hyperuricemia was dysregulated. After JS-2 intervention, SCFAs-producing bacteria were significantly enriched, showing an increase in the numbers of *Ruminococcus_torques_group, Sellimonas, Butyricicoccus, Lachnoclostridium* and *Eubacterium_nodatum_group*. This suggests that SCFA-producing bacteria play a crucial role in the JS-2 treatment of hyperuricemia. SCFAs in quail feces were analyzed using gas chromatography (GC) after two weeks of treatment. The results revealed that the levels of acetic acid, propionic acid, butyric acid, isovaleric acid, and valeric acid in the model group were significantly lower than those in the control group. However, treatment with JS-2 significantly increased the concentrations of these SCFAs compared to the model group and outperformed the positive control drug, benzbromarone (Figure 5).

### 3.5. The Effect of JS-2 on Fecal Metabolites

**Figure 6:**
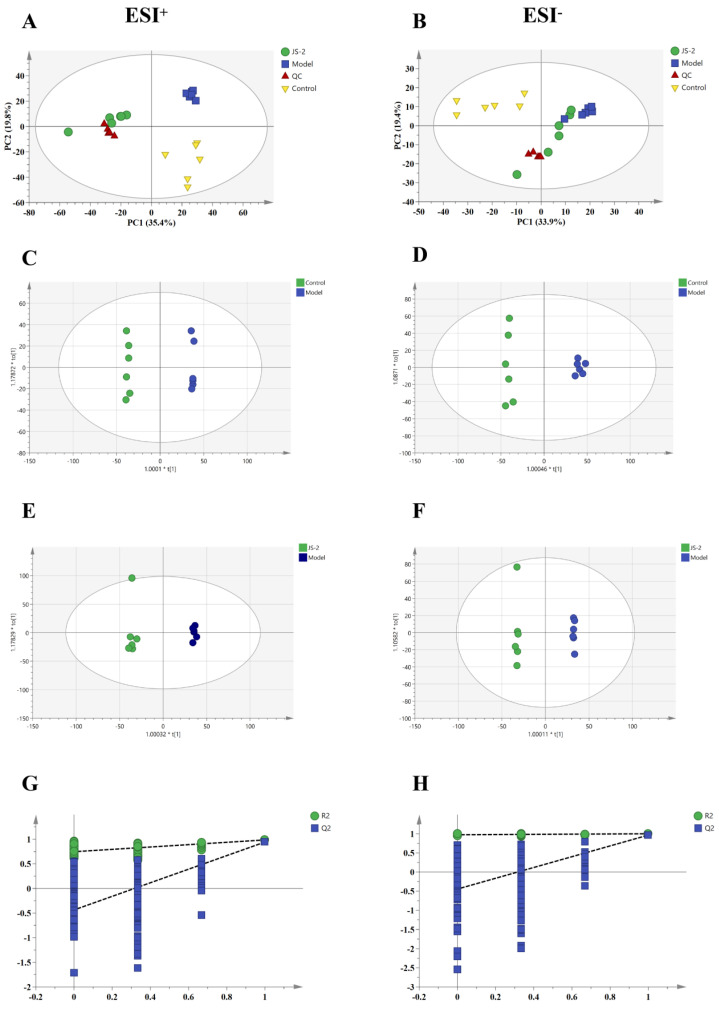
Effects of JS-2 intervention on fecal metabolites in quail models with hyperuricemia. (**A**,**B**) PCA score plot for all groups; (**C**,**D**) OPLS-DA score plot for the control and model groups; (**E**,**F**) OPLS-DA score plot for the model and JS-2 groups; (**G**,**H**) permutation test of OPLS-DA model.

To explore the mechanism of action of JS-2 to meliorate hyperuricemia quails, non-targeted metabolomic analysis was performed on fecal samples. All samples were analyzed by using PCA (model parameters: ESI^+^: R2X = 0.773, Q2 = 0.596; ESI^−^: R2X = 0.753, Q2 = 0.576). The scattered points of each group were distinctly resolved (Figure 6A,B), suggesting that hyperuricemia quails form a distinct fecal metabolic profile, which underwent significant changes after JS-2 intervention. JS-2 intervention partially improved the metabolic disorder in hyperuricemic quails. To further identify biomarkers reflecting significant changes in feces following JS-2 intervention, we compared the control, model, and JS-2 groups using OPLS-DA. The OPLS-DA model (Figure 6C–F) demonstrated a clear separation of the control and JS-2 groups from the model group in both ESI^+^ and ESI^−^ modes. To assess the reliability and potential overfitting of the OPLS-DA model, a 200-permutation test was performed. The test results confirmed that the model was reliable and not overfitting, with R^2^ = 0.746 and Q^2^ = −0.436 in ESI^+^ mode, and R^2^ = 0.974 and Q^2^ = −0.445 in ESI^−^ mode (Figure 6G,H).

The obtained data were integrated with the HMDB database to screen and identify differentially expressed metabolites using the criteria of VIP ≥ 1 and *p* < 0.05, as detailed in Table S2. A total of 20 differential metabolites were identified between the model group and the JS-2 group, with 18 detected in the ESI^+^ mode and 2 in the ESI^−^ mode. JS-2 treatment significantly regulated several metabolites compared to the model group, notably increasing levels of isodesmosine, PE(18:4(6Z,9Z,12Z,15Z)/14:0), ixocarpalactone B and a decrease in fructosyl-lysine, glycogen, styrene oxide, adenine, isoindoline, L-phenylalanine, Val-Val-Val, batrachotoxinin A 20-alpha-benzoate, LysoPC(18:1(9Z)), LysoPI(16:0/0:0), 3-pyridinemethanol, and 6-amino-alpha-(((1-methyl-4-phenylbutyl)amino)methyl). The differential metabolites were introduced into Metabo Analyst 5.0 for metabolic pathway analysis. The differential metabolites in the model and JS-2 groups involved phenylalanine and purine metabolism and biosynthesis of phenylalanine, tyrosine, and tryptophan (Table S2). These findings suggest JS-2 affects amino acid, purine, and phenylalanine metabolism.

### 3.6. Correlations Between Gut Microbiota, Metabolome and Hyperuricemia-Related Parameters

**Figure 7:**
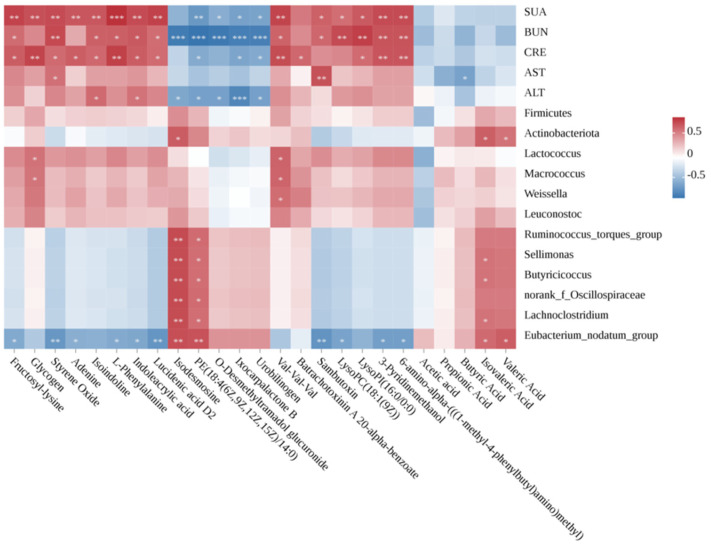
The associations between alterations in the gut microbiota and parameters associated with the metabolome or hyperuricemia were analyzed. The r values are denoted by gradient colors, where red indicates a positive correlation and blue represents a negative correlation. Statistical significance, * *p* < 0.05, ** *p* < 0.01, *** *p* < 0.001.

We employed Spearman correlation analysis to examine the relationships between gut microbiota composition, differential metabolites, and hyperuricemia-related physiological indicators, as shown in Figure 7. The research results indicate that the levels of SUA, BUN, and CRE are most closely related to metabolic differential metabolites, showing a significant correlation. Isodesmosine and PE (18:4(6Z,9Z,12Z,15Z)/14:0) exhibit a significant positive correlation with the microbial community structure formed after JS-2 intervention. The differential metabolites glycogen and Val-Val-Val are significantly positively correlated with the characteristic bacteria in the model group (*Lactococcus*, *Macrococcus* and *Weissella*). Notably, SCFAs, particularly isovaleric acid, are significantly positively correlated with the dominant bacteria in the JS-2 group (*Sellimonas*, *Butyricicoccus*, *Lachnoclostridium* and *Eubacterium_nodatum_group*), while butyric acid is significantly negatively correlated with AST. These correlations emphasize the role of the altered gut microbial structure after JS-2 intervention in regulating differential metabolites and host metabolic parameters.

## 4. Discussion

Hyperuricemia has been becoming more prevalent with each passing year. According to the national survey in 2018–2019, the overall prevalence rate of hyperuricemia among Chinese adults reached 14%. Considerable differences exist in the prevalence of hyperuricemia across diverse regions. The prevalence of hyperuricemia in northwest China (15.5%) is significantly lower than that in northeast China (24.6%) and central China (20.7%), which has attracted our attention [27]. Research suggests that the reduced prevalence of hyperuricemia in northwest China is related to “Jiang Shui”. Jiangshui” is a widely consumed traditional fermented food with a production history of 2000 years and is popular in Shanxi Province and Gansu Province. It contains abundant lactic acid bacteria and other beneficial microbes, contributing positively to gut health by improving the gut microbiota and promoting digestion, absorption, and metabolism. Wu et al. isolated the probiotic fermented Lactobacillus JL-3 strain from “Jiangshui”, which can regulate gut microbiota dysbiosis caused by hyperuricemia and reduce uric acid (UA) levels in mice [22]. Fan et al. isolated the probiotic strain GR4 with high bile salt hydrolase (BSH) activity from “Jiang Shui”, which can effectively control dysregulated bile acids (including conjugated and secondary BAs) to relieve the symptoms of ulcerative colitis (UC) [28]. Zhou et al. isolated the probiotic fermented Lactobacillus GR-3 from “Jiang Shui”, which demonstrates remarkable antioxidant properties through the production of indole derivatives such as indole-3 carboxylic acid (ICA) and pionic acid (IPA). In the AOM/DSS-induced CRC mouse model, GR-3 treatment alleviates weight loss, colon shortening, rectal bleeding, and intestinal barrier damage by reducing oxidative stress and inflammation [29]. At the same time, Han et al. found that GR-3 can restore gut microbiota imbalances induced by As(III) exposure [30]. Many probiotics have demonstrated potential in degrading UA, regulating host metabolism, and preventing chronic diseases and are safer compared with drugs. Our research group previously isolated Lactobacillus paracasei JS-3 and Lactobacillus casei JS-2 from Gansu “Jiang shui”. Among them, JS-3 can improve hyperuricemia by modulating the gut microbiota and its associated metabolism [31]. This study will continue to explore the ability of JS-2 to lower hyperuricemia and provide candidate strains for the treatment of hyperuricemia.

In this research, the untreated ‘Jiangshui’ sample was grown in a medium using uric acid as the sole carbon and nitrogen source. A strain possessing excellent uric acid degradation capabilities was isolated and identified as Lactobacillus casei JS-2. JS-2 has shown a significant ability to reduce uric acid in vitro within 24 h and can degrade 23.05% of uric acid within 72 h. JS-2 has good growth characteristics, reaching its growth peak at 24 h and then entering the decline phase. JS-2 can survive under acidic conditions and has a high acid-producing ability. The pH value decreased by two orders of magnitude at 24 h and it can survive smoothly in simulated gastrointestinal fluids. To further evaluate the ability of JS-2 to degrade uric acid, we selected quails that are similar to humans and lack uricase. An appropriate hyperuricemia model based on a high-purine diet was employed to closely mimic the clinical scenario and assess the in vivo UA degradation ability of JS-2. The results showed that JS-2 can significantly reduce the serum uric acid level of hyperuricemic quails and is superior to the positive drug benzbromarone. In addition, JS-2 also improved the liver and kidney damage caused by hyperuricemia in quails. After two weeks of JS-2 treatment, the levels of BUN, CRE, and AST showed a significant reduction compared to the model group. Through pathological sections, it can be observed that there was inflammatory cell infiltration in kidney tissue, degeneration or necrosis of renal tubular epithelial cells, accompanied by nuclear pyknosis or karyolysis, cytoplasmic vacuolation, and mild interstitial edema. Despite its effectiveness, the precise mechanism underlying JS-2′s ability to degrade uric acid has yet to be clarified.

Research indicates that approximately two-thirds of uric acid is eliminated by the kidneys, while the remainder is either excreted in feces or metabolized by the gut microbiota. The functional impairment resulting from hyperuricemia directly impacts the elimination of UA. Studies have found that there is an imbalance in the gut microbiota of hyperuricemic patients [32,33]. In hyperuricemic patients, the microbiota exhibits reduced richness and diversity compared to healthy individuals, its composition is altered, and the relative abundance of Coprococcus is lower [34]. Probiotics have the effect of reducing UA and are capable of altering the gut microbiota [35]. Since JS-2 can colonize in the intestine, we collected the feces of quails from different groups and cultured them with GAM medium for 24 h to evaluate the ability of fecal microbiota to degrade UA. The findings revealed that the fecal microbiota in the JS-2 group demonstrated a strong capacity for uric acid degradation. The positive impact of JS-2 on the intestines of hyperuricemic quails has sparked interest in investigating the relationship between the gut microbiota, its metabolites, and hyperuricemia. Therefore, we conducted 16S rRNA sequencing analysis on the feces of quails in each group to evaluate the changes in the systemic gut microbiota driven by JS-2. The findings revealed a significant reduction in gut microbial diversity in hyperuricemic quails, while JS-2 treatment corrected gut dysbiosis, facilitated the recovery of healthy microbial structures, and markedly enhanced ecosystem diversity. Compared with the normal group, the phylum Firmicutes and the phylum Actinobacteriota in the model group were significantly increased and were improved after 2 weeks of JS-2 intervention. Lactococcus, Micrococcus, Weissella, and Leuconostoc constituted the unique gut microbiota structure of hyperuricemic quails, and their abundances were significantly decreased after JS-2 intervention. In addition, the quails in the JS-2 group formed a special gut microbiota structure, with the abundances of Ruminococcus_torques_group, Sellimonas, Butyricicoccus, norank_f_Oscillospiraceae, Lachnoclostridium, and Eubacterium_nodatum_group being significantly increased.

Disruptions in the gut microbiota can also severely impact the metabolic levels of the body. Fecal metabolomics analysis has shown that hyperuricemic quails present severe metabolic disturbances. Metabolites and their derivatives are either expelled through feces or absorbed into the body, causing a range of biological outcomes. Given that JS-2 possesses a significant acid-producing ability and can enrich the microbiota capable of producing short-chain fatty acids (SCFAs), such as Ruminococcus_torques_group, Sellimonas, Butyricicoccus, Lachnoclostridium, and Eubacterium_nodatum_group, we measured the content of SCFAs in feces, as they are among the crucial metabolites of the gut microbiota. SCFAs play a direct role in metabolic health by influencing tissue-specific processes, including appetite control, energy balance, glucose regulation, and immune function. Gut microbiota-derived SCFAs, acting as anti-inflammatory mediators, can even improve obesity and reduce insulin resistance [36]. Our research findings reveal that the levels of SCFAs in hyperuricemic quails are significantly decreased, and this situation is notably improved after JS-2 treatment, especially for acetic acid, propionic acid, and valeric acid. Correlation analysis shows that isovaleric acid is significantly correlated with Sellimonas, Butyricicoccus, Lachnoclostridium, and Eubacterium_nodatum_group, while valeric acid is significantly correlated with Eubacterium_nodatum_group. After JS-2 intervention, some abnormal metabolites are markedly reversed, involving the biosynthetic pathways of phenylalanine, tryptophan, tyrosine, and adenine. The changes in these metabolites are closely associated with hyperuricemia. Notably, the serum levels of uric acid (SUA), blood urea nitrogen (BUN), and creatinine (CRE) are most closely and significantly correlated with the differentially metabolized metabolites. This suggests that alterations in the gut environment impact key metabolic pathways, with interventions targeting purine metabolism being particularly crucial for managing hyperuricemia. There are numerous methods for establishing HUA animal models, which are commonly seen in poultry and rodents [37]. Currently, mice are the most frequently used; however, they express uricase, which can break down UA into allantoin for excretion. Quails do not contain urate oxidase and are an ideal model for HUA, as their uric acid metabolism is physiologically consistent with humans [3,31]. In this study, Lactobacillus casei with UA degradation ability was screened from “Jiangshui” to evaluate the effect of the selected strain on a quail model for HUA developed using yeast extract. Meanwhile, we also conducted an in-depth study on the relationships among the gut microbiota, fecal metabolites, and HUA and explored the mechanism of action.

## 5. Conclusions

In this study, we discovered a strain, Lactobacillus casei JS-2, with UA degradation ability from “Jiangshui”. This study utilized quails as an animal model to investigate UA degradation. JS-2 can reduce the UA level in quails mainly by restoring the diversity of the gut microbiota and increasing the abundance of SCFA-producing and UA-related bacteria. JS-2 enhances the microbial diversity and functionality in hyperuricemic quails by enriching SCFA-producing bacteria such as Ruminococcus_torques_group, Sellimonas, Butyricicoccus, and Lachnoclostridium. Fecal metabolites indicate that JS-2 regulates purine metabolism by reducing adenine and inhibiting UA production.

However, our study still has limitations. While JS-2 is capable of surviving in the gastrointestinal tract, its presence within the gut microbiota has not yet been confirmed. Secondly, the regulatory mechanism of JS-2 must be further elucidated, as human metabolic processes may differ from those of animal models. Clinical trials are needed to evaluate the therapeutic efficacy of this strain. Meanwhile, future studies should adopt long-term experimental designs and multi-omics analyses to assess the long-term safety and potential side effects of JS-2. Overall, Lacticaseibacillus casei JS-2 offers a straightforward and efficient approach to treating hyperuricemia, providing a promising therapeutic strategy for the biotherapy of HUA.

## Data Availability

The original contributions presented in this study are included in the article/Supplementary Materials. Further inquiries can be directed to the corresponding author.

## Supplementary Materials

The following supporting information can be downloaded at: https://www.mdpi.com/article/10.3390/foods14030407/s1, Figure S1: Rarefaction curve (A) Sobs index. (B) Shannon index. (C) Simpson index; Table S1: Survival rate of JS-2 in artificial gastric and intestinal fluids; Table S2: Potential biomarker information. The up (↑) and down (↓) arrows represent the relative increasing or decreasing trend of the metabolites.

## Abbreviations

UA, uric acid; HUA, hyperuricemia; XOD, xanthine oxidase; SEM, scanning electron microscope; CRE, creatinine, BUN, blood urea nitrogen; SCFAs, short-chain fatty acids; OTUs, operational taxonomic units; PCoA, principal coordinate analysis.
