# Supplementary material for: Lacticaseibacillus casei JS-2 from ‘Jiangshui’ Reduces Uric Acid and Modulates Gut Microbiota in Hyperuricemia
Source: Foods. 2025 Jan 26;14(3):407. doi: 10.3390/foods14030407 (PMC11817023; doi:10.3390/foods14030407)
Supplement: Supplementary file 1 [file foods-14-00407-s001.zip › foods-3406725-tables.pdf]

Table S1 Survival rate of JS-2 in artificial gastric and intestinal fluids

simulated liquid	artificial gastric juice	simulated intestinal fluid	
	3h	4h	8h
PH=2	50.56±1.32	30.97±7.39	34.33±0.53
PH=3	82.09±16.73	76.37±13.68	60.57±16.74
PH=4	98.26±10.77	95.52±5.02	88.06±9.33

Table S2: Potential biomarker information. The up (↑) and down (↓) arrows represent the relative increasing or decreasing trend of the metabolites.

No.	**Rt/ （min）**	***m/z***	**metabolites**	**Chemical formula**	**mode**	JS3 vs Model
1	0.705	309.16571	Fructosyl-lysine	$C_{12}H_{25}N_2O_7$	$[M+H]^+$	↓
2	0.726	689.21088	Glycogen	$C_{23}H_{33}N_{10}O_{15}$	$[M+Na]^+$	↓
3	1.27	121.06478	Styrene Oxide	C_8H_8O	$[M+H]^+$	↓
4	1.355	136.0618	Adenine	$C_5H_5N_5$	$[M+H]^+$	↓
5	2.197	120.08064	Isoindoline	$C_8H_{10}N$	$[M+H]^+$	↓
6	2.231	166.08632	L-Phenylalanine	$C_9H_{12}O_2N$	$[M+H]^+$	↓
7	4.845	188.07059	Indoleacrylic acid	$C_{11}H_9NO_2$	$[M+H]^+$	↓
8	7.545	515.26202	Lucidenic acid D2	$C_{29}H_{38}O_8$	$[M+H]^+$	↓
9	8.228	526.29089	Isodesmosine	$C_{24}H_{40}N_5O_8$	$[M+H]^+$	↑
10	9.144	701.39264	PE(18:4(6Z,9Z,12Z,15Z)/14:0)	$C_{37}H_{66}NO_8P$	$[M+NH_4]^+$	↑
11	9.44	426.21439	O-Desmethyltramadol glucuronide	$C_{21}H_{31}NO_8$	$[M+H]^+$	↑
12	10.438	503.26459	Ixocarpalactone B	$C_{28}H_{38}O_8$	$[M+H]^+$	↑

13	10.902	591.31683	Urobilinogen	$C_{33}H_{42}N_4O_6$	$[M+H]^+$	↑
14	12.747	333.25107	Val-Val-Val	$C_{15}H_{33}N_4O_4$	$[M+NH_4]^+$	↓
15	13.506	544.26727	Batrachotoxinin A 20-alpha-benzoate	$C_{31}H_{39}NO_6Na$	$[M+Na]^+$	↓
16	18.328	454.2923	Sambutoxin	$C_{28}H_{39}NO_4$	$[M+H]^+$	↓
17	19.006	522.35413	LysoPC(18:1(9Z))	$C_{26}H_{53}O_7NP$	$[M+H]^-$	↓
18	20.062	571.28778	LysoPI(16:0/0:0)	$C_{25}H_{49}O_{12}P$	$[M-H]^-$	↓
19	23.641	317.23288	3-Pyridinemethanol	$C_{18}H_{29}N_4O$	$[M+NH_4]^+$	↓
20	23.641	317.23288	6-amino-alpha-(((1-methyl-4-phen ylbutyl)amino)methyl)	$C_{18}H_{29}N_4O$	$[M+NH_4]^+$	↓
