# Supplementary figures and images for: Lacticaseibacillus casei JS-2 from ‘Jiangshui’ Reduces Uric Acid and Modulates Gut Microbiota in Hyperuricemia
Source: Foods. 2025 Jan 26;14(3):407. doi: 10.3390/foods14030407 (PMC11817023; doi:10.3390/foods14030407)

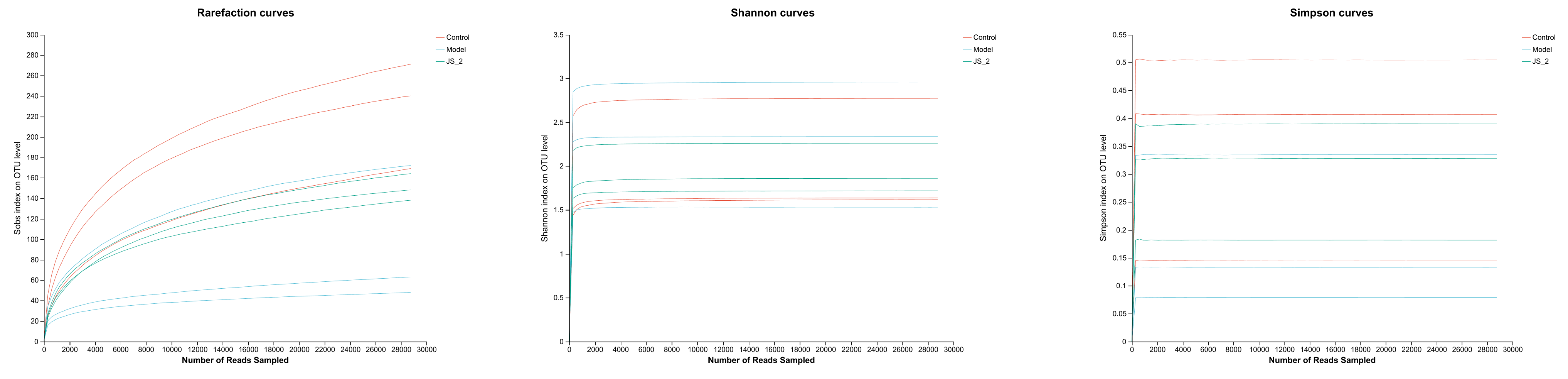

Supplement: Supplementary file 1 [file foods-14-00407-s001.zip › Figure S1.tif]
